# Kushenin Combined with Adefovir Dipivoxil or Entecavir for Chronic Hepatitis B: A Systematic Review and Meta-Analysis

**DOI:** 10.1155/2021/8856319

**Published:** 2021-02-25

**Authors:** Qingying Liao, Jianxia Wen, Kunxiu Jiang, Yanling Zhao, Xiao Ma

**Affiliations:** ^1^State Key Laboratory of Southwestern Chinese Medicine Resources, School of Pharmacy, Chengdu University of Traditional Chinese Medicine, Chengdu 611137, China; ^2^Department of Pharmacy, The Fifth Medical Center of PLA General Hospital, Beijing 100039, China; ^3^School of Pharmacy, Beijing University of Traditional Chinese Medicine, Beijing 102488, China

## Abstract

Kushenin (KS) has become a traditional Chinese medicine preparation that plays an important role in treating chronic hepatitis B (CHB). Many clinical studies have discussed its curative effect and safety in combination with adefovir dipivoxil (ADV) or entecavir (ETV) for treating CHB, but there is still a lack of a systematic analysis. Therefore, this study evaluated the efficacy and safety of KS through a meta-analysis to better guide clinical treatment. Seven databases were searched to identify randomized controlled trials (RCTs) concerning KS combined with ADV or ETV for treating CHB. The primary outcomes included serum viral indices and adverse events, and the secondary outcomes were liver function indices. The risk of bias of the included RCTs was appraised by Cochrane software. STATA 15.1 and Review Manager 5.3 software were used for the meta-analysis. Thirty-two RCTs recruiting 3343 patients with CHB were collected for this meta-analysis. KS combined with ETV or ADV led to an amelioration of the CHB index to various degrees. In short, the meta-analysis indicated that the combination group, compared to the single group, showed great improvement in HBeAg seroconversion, frequency of undetectable HBV-DNA levels, loss of serum HBeAg, and loss of serum HBsAg. The combination treatment also decreased serum HBV-DNA levels when compared to the levels after the single treatment. However, KS combined with ADV or ETV displayed no remarkable difference in the incidence of adverse events or in serum ALT levels. Current evidence showed that, compared with the use of either drug alone, KS combined with ADV or ETV can improve the clinical efficacy of CHB treatment.

## 1. Introduction

Chronic hepatitis B (CHB), which is one of the most significant global health issues, seriously endangers the lives of humans worldwide. The number of people in the world who are chronically infected with hepatitis B virus exceeds 350 million, accounting for approximately 5% of the world's population [1]. Hepatitis B virus, which is a deoxyribonucleic acid virus of the hepadnaviridae family, only replicates in human hepatocytes and causes serious damage to human hepatocytes [2]. When human beings are infected with hepatitis B virus, if they do not receive effective treatment, the disease may slowly develop into liver fibrosis, cirrhosis and even hepatocellular carcinoma [3]. Therefore, it is very important to seek effective treatment for CHB to ensure a healthy life.

At present, there are mainly two methods used to treat CHB. One method is to take antiviral drugs, such as nucleoside analogues, which can prevent the release of infectious viruses; the other method is to use conventional or pegylated IFN-*α* to stimulate the patient's antiviral immune response [2]. However, antiviral therapy, which is the use of antiviral drugs such as nucleoside/nucleotide analogues to treat patients with CHB, not only has a high relapse rate after drug withdrawal but also promotes the development of drug resistance in the virus, thus accelerating the deterioration caused by the disease. In addition, immunomodulators such as conventional or polyethylene glycol interferon have very limited curative effects, and some side effects, such as flu-like symptoms, may occur during the treatment process [2, 4]. Consequently, we still need to find a safer and more effective therapy for CHB.

China is a region with a high incidence of CHB and has a long history using traditional Chinese medicine (TCM) to treat CHB, leading to increasingly improved treatment concepts and clinical experience [5]. Currently, TCM is still widely used to help patients with CHB in China and other countries. For example, some clinical studies show that more than 80% of the natural products considered helpful for liver diseases are Chinese herbal medicines or their extracts [6].

Consequently, therapy in which TCM is integrated with modern medicine has gradually become an important way to treat CHB. For example, Kushenin (KS) combined with adefovir dipivoxil (ADV) or entecavir (ETV) for CHB patients has an obvious curative effect, as confirmed in many clinical studies. However, an analysis of its curative effect and application characteristics from the perspective of evidence-based pharmacy is still lacking. To provide possibly better alternative therapies for global application, this study presented a meta-analysis of KS combined with ADV or ETV for patients with CHB and evaluated its efficacy through indicators such as serum liver fibrosis, serum viral indices, liver function index, and adverse events.

## 2. Materials and Methods

### 2.1. Research Registration

This research programme has been registered in PROSPERO, and the PROSPERO registration number is CRD42019138487.

### 2.2. Ethical Approval and Consent to Participate

As the current study does not involve animal experiments and patient consent, the ethics approval and consent to participate are not applicable.

### 2.3. Database and Search Strategy

The following English and Chinese databases were comprehensively searched by two researchers: China National Knowledge Infrastructure (CNKI), PubMed, Chinese Biomedical Database, Wanfang, Embase, Cochrane Library, and VIP medicine information system. The dates ranged from the inception of the databases to Sep 2020. The search strategy included only terminologies related to the intervention. Search terms included “Kushenin,” “chronic hepatitis B,” “adefovir dipivoxil,” and “entecavir,” and the search strategies were adjusted in different databases.

### 2.4. Inclusion Criteria

Four main inclusion criteria are listed as follows. (1) The studies were randomized controlled trials (RCTs) that were published in Chinese or English. (2) The diagnostic criteria of the patients with CHB conformed to those outlined in the “Viral Hepatitis Prevention Plan” or the “Guidelines for the Prevention and Treatment of Chronic Hepatitis B,” and the hepatitis B e antigen (HBeAg) test was positive. (3) Subjects in the control group take only ADV or ETV. Patients in the experimental group take KS combined with ADV or ETV, and the administration route must be oral. The treatment course of the experimental group must be the same as that of the control group. (4) Studies with sufficient objective results were selected for this analysis. The primary outcomes of this review were serum viral indices and adverse events, and the secondary outcomes of this review included the liver function indices. The measured results in the current study were all based on reference to a certain period after the end of treatment. The included trials should report at least one of the aforementioned outcomes.

### 2.5. Exclusion Criteria

Studies meeting any of the following criteria were excluded from the analysis: (1) studies in which the subjects presented with severe symptoms, such as liver failure and liver cirrhosis or complications with other viral hepatitis diseases, autoimmune hepatitis, drug-induced hepatitis, hereditary liver disease, and so on, (2) non-RCTs such as literature reviews, comments, and animal experiments, (3) nonoriginal research or duplicate publications, (4) studies with missing data or incorrect data, and (5) literature with poor research quality or insufficient evaluation of outcomes.

### 2.6. Data Extraction

Two researchers searched the aforementioned Chinese and English databases, according to the retrieval strategy to obtain studies that may meet the inclusion criteria. Then, they read the full text of these articles and decided on the final studies to be included according to the inclusion and exclusion criteria. Data extraction was executed independently by two other researchers. Extracted information included baseline information of patients, intervention and control measures, outcome data, and other information, and these detailed data were imported into Cochrane Review Manager 5.3 to be prepared for further analysis. Missing data were requested from the corresponding authors.

### 2.7. Risk of Bias Assessment

A quality assessment of the included RCTs was carried out independently by two researchers with the use of the Cochrane risk of bias tool, which is a common tool for evaluating methodological quality. This tool has seven aspects, including blinding of participants and personnel (performance bias), random sequence generation (selection bias), blinding of outcome data (attrition bias), allocation concealment (selection bias), selective reporting (reporting bias), incomplete outcome data (attrition bias), and other sources of bias. Finally, three evaluation outcomes were noted for each aspect: high risk, low risk, and unclear risk. Studies that satisfied all criteria were classified as low risk. Trials that did not meet any criteria were classified as high risk. Studies were classified as unclear risk of bias if there was not enough information to make a judgment. Any differences arising in the process of study retrieval, data extraction, and quality assessment were settled through discussion and negotiation with another researcher.

### 2.8. Data Analysis

All data analyses were carried out by using STATA 15.1 and Cochrane Review Manager 5.3. Relative risk (RR) was used for binary variables, and standardized mean difference (SMD) was used for continuous variables. Heterogeneity between the studies in effect measures was assessed using both the *P* test and the *I*^*2*^ statistic. In detail, the *I*^*2*^ value greater than 50% and the *P* value less than 0.01 were considered indicative of substantial heterogeneity, at which point the random effect model was used; otherwise, the fixed effect model was adopted.

Publication bias was evaluated by funnel plot analysis. If scatter points were symmetrically distributed on both sides of the funnel, the possibility of publication bias was small; otherwise, the possibility of publication bias was large. If the necessary data were available, subgroup analyses were carried out according to different treatment period.

## 3. Results

### 3.1. Characteristics of Included Trials

**Figure 1:**
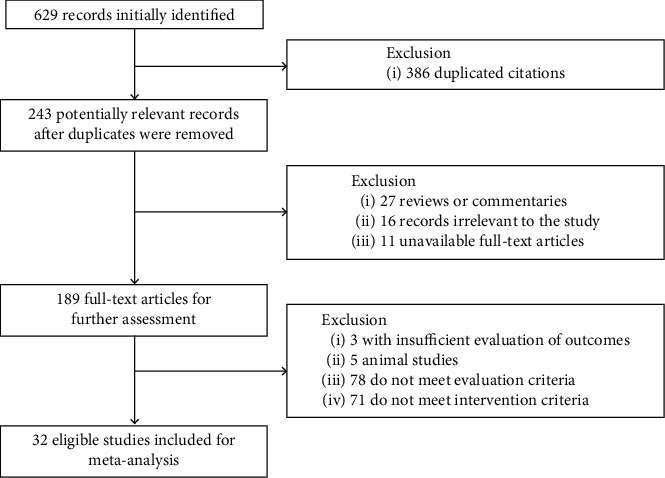
Flowchart of research selection.

In total, 629 related studies were retrieved through the database search. Following the removal of 386 duplicated citations, 243 potentially relevant records were reserved. Next, 54 studies with comments and irrelevant to the study were further removed. Full-text articles of 189 publications were evaluated for further assessment. Among them, trials with insufficient evaluation of outcomes, animal studies, and trials not meeting evaluation and intervention criteria were excluded. Finally, a total of 32 trials recruiting 3343 patients were included for subsequent meta-analysis after reading the full-text [7–38] (Figure 1). Of these 32 articles, 18 articles included subjects treated with KS and ADV [7–24], and 14 articles included subjects treated with KS and ETV [25–38]. The control group was treated with ADV (10 mg/d, po, qd) or ETV (0.5 mg/d, po, qd) alone. Most of the experiment group was given KS (0.2 g/time, po, tid) [7–9, 11–14, 16, 18–21, 23–38] on the basis of the control group, and several studies used different doses of KS, 0.15 g/time, po, tid [17], 0.3 g/time, po, tid [10, 15], and 0.4 g/time, po, tid [22]. All drugs were administered orally. No significant differences appeared in the age, course of disease, or sex between the two groups, and fourteen studies reported slight adverse events (Table 1).

### 3.2. Methodological Quality of Included Trials

**Figure 2:**
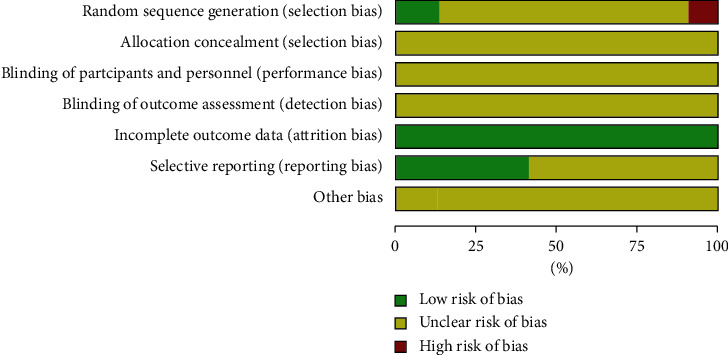
Methodological quality assessment of the included studies. Red square indicates a high risk of bias, green square indicates low risk of bias, and blank square indicates unclear risk of bias.

The methodological quality of the 32 included RCTs was evaluated and is presented in Figure 2. All trials were described as RCTs, of which five trials described the randomization method in detail [8, 17, 30, 31, 35]. Four trials [17, 31, 37, 38] adopted the random number table method and were considered to be of low risk of selection bias as the patients were randomly divided into two groups. All RCTs had complete data. However, the allocation concealment, blinding of participants and personnel, other bias reports, and blinding of outcome assessments were unclear in all trials. At the same time, the selective reporting of most studies was unclear, with only 10 studies considered to be of low risk with respect to reporting bias [8–11, 15, 16, 19, 25, 26, 31] (Figure 2).

### 3.3. Outcome Measures

#### 3.3.1. Undetectable Serum HBV-DNA Rate

**Figure 3:**
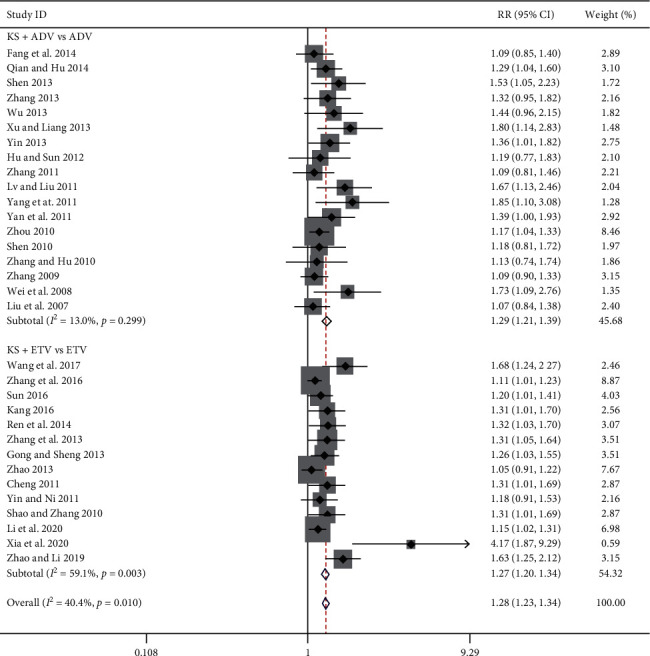
Forest plot of undetectable serum HBV-DNA rate in CHB patients treated with KS combined with ADV or ETV.

Thirty-two studies reported the undetectable serum HBV-DNA rate [7–38], 18 of which were in the KS + ADV group and the other 14 were in the KS + ETV group. Meta-analysis results showed that, compared with the ADV group, the KS + ADV group presented a significant improvement in the undetectable serum HBV-DNA rate (RR = 1.29, 95% CI (1.21, 1.39), *P* < 0.00001) (*I*^2^ = 13.0%, *P*=0.30). Compared with the ETV group, the KS + ETV group also showed a significant improvement in the undetectable serum HBV-DNA rate [RR = 1.27, 95% CI (1.20, 1.34), *P* < 0.00001] (*I*^2^ = 59%, *P*=0.003) (Figure 3).

#### 3.3.2. Loss of Serum HBeAg Rate

**Figure 4:**
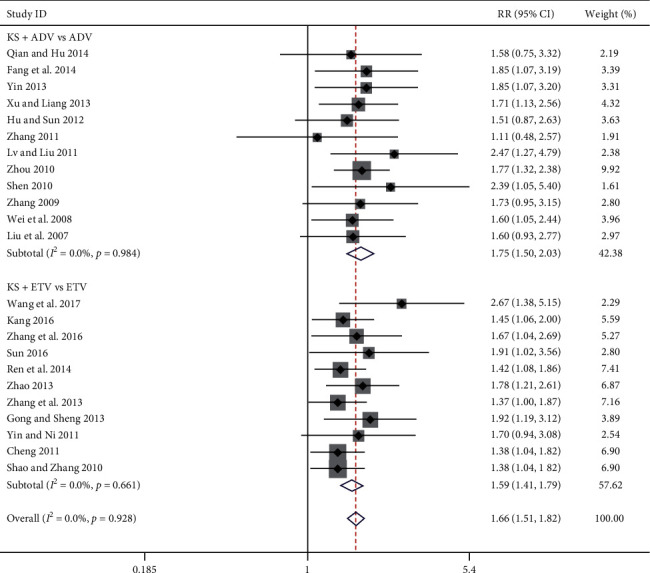
Forest plot of KS combined with ADV or ETV on loss of serum HBeAg rate.

Twenty-three studies [7–12, 14, 16, 19, 20, 23–35] reported the loss of serum HBeAg rate, 12 of which were in the KS + ADV group and the other 11 were in the KS + ETV group. Meta-analysis results showed that, compared with the ADV group, the KS + ADV group presented a significant improvement in the rate of loss of serum HBeAg [RR = 1.75, 95% CI (1.50, 2.03), *P* < 0.00001] (*I*^2^ = 0.0%, *P*=0.98). Compared with the ETV group, the KS + ETV group also showed a significant improvement in the loss of serum HBeAg rate [RR = 1.59, 95% CI (1.41, 1.79), *P* < 0.00001] (*I*^*2*^ = 0.0%, *P*=0.66) (Figure 4).

#### 3.3.3. HBeAg Seroconversion Rate

**Figure 5:**
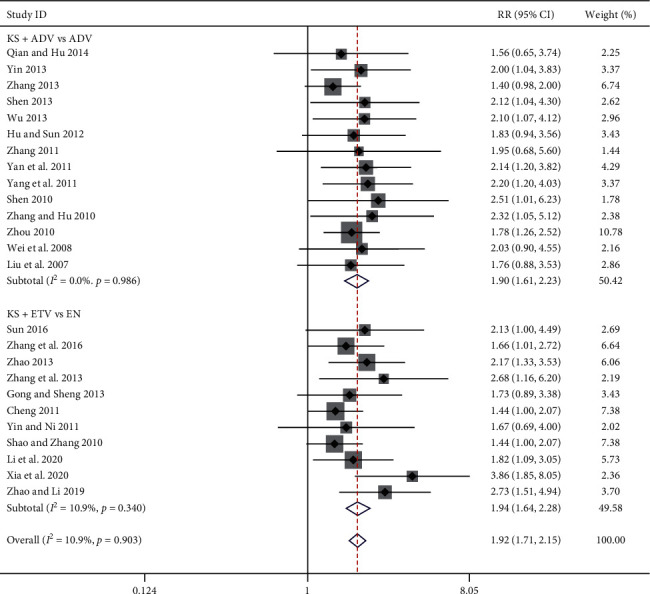
Forest plot of HBeAg seroconversion rate in patients treated with KS combined with ADV or ETV.

Twenty-five studies reported the HBeAg seroconversion rate [8, 9, 11–15, 17–19, 21–26, 29, 30, 32–38], 14 of which belong to the KS + ADV group and the other 11 belong to the KS + ETV group. The meta-analysis results showed that, compared with the ADV group, the KS + ADV group had a significant improvement in the HBeAg seroconversion rate [RR = 1.90, 95% CI (1.61, 2.23), *P* < 0.00001] (*I*^2^ = 0.0%, *P*=0.99). Compared with the ETV group, the KS + ETV group also showed a significant improvement in the HBeAg seroconversion rate [RR = 1.94, 95% CI (1.64, 2.28), *P* < 0.00001] (*I*^2^ = 11%, *P*=0.34) (Figure 5).

#### 3.3.4. Loss of Serum HBsAg Rate

**Figure 6:**
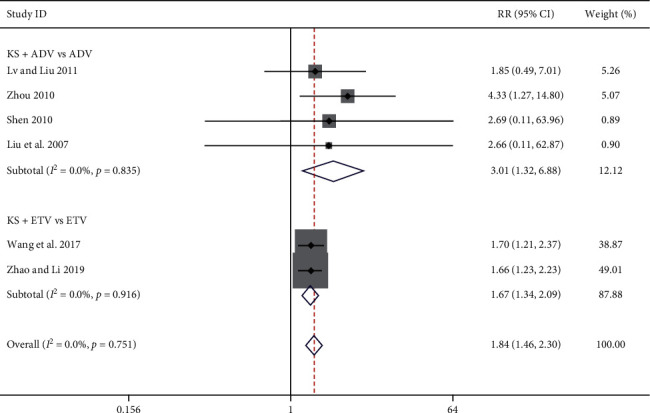
Forest plot of KS combined with ADV or ETV on loss of serum HBsAg rate.

Six studies reported the loss of serum HBsAg rate [9, 10, 12, 24, 31, 38], four of which were in the KS + ADV group and the other two were in the KS + ETV group. The meta-analysis results show that, compared with the ADV group, the KS + ADV group presented a significant improvement in the loss of serum HBsAg rate [RR = 3.01, 95% CI (1.32, 6.88), *P*=0.009] (*I*^2^ = 0.0%, *P*=0.84). Compared with the ETV group, the KS + ETV group also showed a significant improvement in the loss of serum HBsAg rate [RR = 1.67, 95% CI (1.34, 2.09), *P* < 0.00001] (*I*^*2*^ = 0.0%, *P*=0.75) (Figure 6).

#### 3.3.5. ALT Normalization Rate and Serum ALT Levels

**Figure 7:**
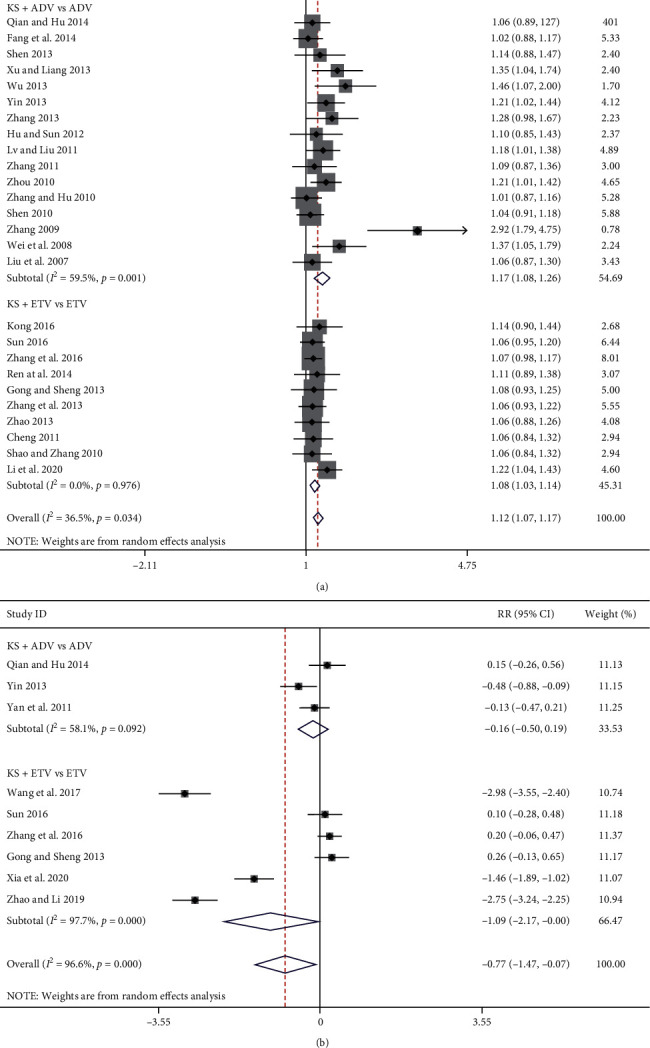
Forest plot of ALT normalization rate and serum ALT levels in CHB patients treated with KS combined with ADV or ETV. (a) ALT normalization rate. (b) Serum ALT levels.

Twenty-six studies reported the ALT normalization rate [7–16, 19–30, 33–36], 16 of which were in the KS + ADV group and the other 10 were in the KS + ETV group. The meta-analysis results show that, compared with the ADV group, the KS + ADV group had a significant improvement in the ALT normalization rate [RR = 1.17, 95% CI (1.08, 1.26), *P*=0.0001] (*I*^*2*^ = 60%, *P*=0.001) (Figure 7(a)). Compared with the ETV group, the KS + ETV group also showed a significant improvement in the ALT normalization rate [RR = 1.08, 95% CI (1.03, 1.14), *P*=0.003] (*I*^*2*^ = 0.0%, *P*=0.57) (Figure 7(a)).

Nine studies reported serum ALT levels [11, 17, 19, 26, 30, 31, 34, 37, 38], three of which were in the KS + ADV group and the other six were in the KS + ETV group. The meta-analysis results indicate that, compared with ADV, KS + ADV did not show a significant effect on the serum ALT levels [SMD = −0.16, 95% CI (−0.50, 0.19), *P*=0.37] (*I*^*2*^ = 57%, *P*=0.10). Compared with ETV, KS + ETV also showed no significant effect on the serum ALT levels [SMD = −1.09, 95% CI (−2.17, 0.00), *P*=0.05] (*I*^*2*^ = 97%, *p* < 0.00001) (Figure 7(b)).

#### 3.3.6. Serum HBV-DNA Levels

**Figure 8:**
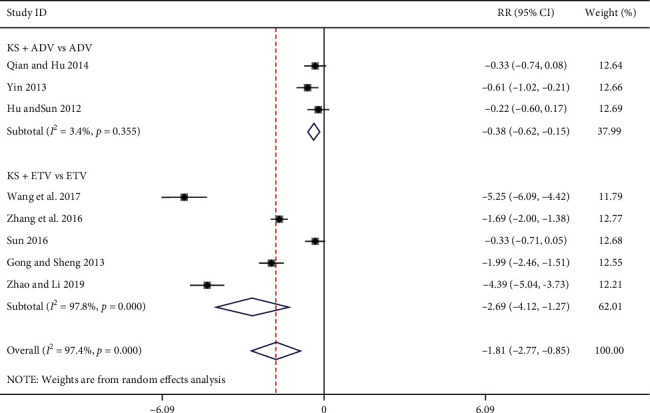
Forest plot of serum HBV-DNA levels in CHB patients treated with KS combined with ADV or ETV.

Eight studies reported serum HBV-DNA levels [8, 11, 19, 26, 30, 31, 34, 38], three of which were in the KS + ADV group and the other five were in the KS + ETV group. The meta-analysis results show that, compared with the ADV group, the KS + ADV group experienced a significant reduction in serum HBV-DNA levels (SMD = −0.38, 95% CI (−0.62, −0.15), *P*=0.001) (*I*^*2*^ = 2%, *P*=0.36) (Figure 8(a)). Compared with the ETV group, the KS + ETV group also showed a significant reduction in serum HBV-DNA levels [SMD = −2.69, 95% CI (−4.12, −1.27), *P*=0.0002] (*I*^*2*^ = 97%, *p* < 0.00001) (Figure 8(b)).

### 3.4. Adverse Events

Twenty-one studies reported on adverse reactions [7–11, 15–19, 21–24, 26–28, 30, 33, 37, 38], of which four mentioned that there were no obvious adverse reactions in either the single drug group or the combined drug group [9, 10, 15, 27]. The other 17 studies mentioned that patients experienced mild adverse reactions, mainly including nausea, vomiting, anorexia, acid regurgitation, abdominal discomfort, diarrhoea, dizziness, headache, fever, fatigue, chest tightness, skin pruritus, rash, creatinine elevation, proteinuria, and transient decrease in cholinesterase. All adverse reactions were mild, could be tolerated by patients, and gradually disappeared after symptomatic treatment or no treatment. Although there was no significant difference in the incidence of adverse reactions between the trial group and the control group, further investigation is still needed to carry out a systematic safety evaluation of the combination of KS and ETV or ADV (Supplemental Digital Content).

### 3.5. Subgroup Analysis on Different Courses of Treatment

Considering that different courses of treatment may affect the magnitude of the drug treatment effect, this study carried out a subgroup analysis according to the courses of treatment to more comprehensively appraise the curative effect of KS combined with ADV or ETV. As shown in Table 2, undetectable serum HBV-DNA rate, loss of serum HBeAg rate, HBeAg seroconversion rate, and ALT normalization rate less than one year and one year or more were analyzed in the forms of subgroup. The results indicated that, compared with the single drug group, KS combined with ADV or ETV was more effective in terms of loss of serum HBeAg rate, HBeAg seroconversion rate, and ALT normalization rate while the treatment period was greater than or equal to one year. However, in less than one year, the undetectable serum HBV-DNA rate improved significantly. These results indicated that the potential publication bias still existed.

## 4. Discussion

In China, TCM treatment has always been an important method to prevent and treat CHB. For example, KS is a representative antiviral TCM preparation and has a potential antivirus effect. Modern pharmacological studies show that KS has certain antiviral, anti-inflammatory and antitumour effects, and it has attracted much attention in the treatment of CHB [39]. KS is a pure natural alkaloid drug separated from the root of *Sophora alopecuroides* and *Sophora flavescens* in Leguminosae. It is a mixed alkali of oxymatrine and a very small amount of oxysophocarpine, of which oxymatrine accounts for more than 98% and is the main component of KS [40]. The mechanism of action of KS is to inhibit the damage to hepatocytes caused by the hepatitis virus by promoting the expression of microRNA-122 and interferon-*α* in hepatocytes. Moreover, the anti-HBV effect of KS is produced by blocking the adsorption of hepatitis virus and entry into cells, inhibiting the expression of hepatocytes and secreting HBsAg, HBeAg, and HBV-DNA [41]. Our previous study [42] had assessed the clinical efficacy as well as the safety of KS combined with nucleoside analogues (NAs), including lamivudine (LAM), ADV, ETV, and telbivudine (TLV) for the treatment of CHB. The results indicated that the combination of KS and NAs improves the clinical efficacy of NAs in CHB with no obvious adverse effect. In this study, the literatures were updated, and a more detailed analysis of KS combined with ADV or ETV for the treatment of CHB was performed. Simultaneously, the loss of serum HBsAg rate and serum HBV-DNA levels of KS combined with ADV or ETV for CHB was analyzed, and a subgroup analysis of different courses of treatment was performed. However, due to the methodological quality of the included studies, more rigorous, large sample and well-designed RCTs are needed to confirm these findings.

This study had further confirmed the clinical efficacy of KS combined with ADV or ETV in the treatment of CHB from the perspective of evidence-based pharmacy through a meta-analysis. Thirty-two RCTs involving 3343 people with CHB were ultimately included. This meta-analysis showed that KS combined with ADV or ETV was effective in treating CHB, and this efficacy was manifested by different changes in various indicators of CHB. Compared with the single agents, the KS combined with ADV or ETV groups not only improved ALT normalization, loss of serum HBeAg, loss of serum HBsAg, HBeAg seroconversion, and undetectable serum HBV-DNA rates but also reduced serum HBV-DNA levels to a certain extent. These findings indicated that patients are transitioning to a much lower HBV replication state and degree of liver injury. No serious adverse reactions occurred in any of the included studies. Even though minor adverse reactions occurred, they were tolerated by patients and gradually disappeared, which showed that KS combined with ADV or ETV was very safe. Notably, there were no significant differences in side effects or serum ALT levels between the combined drug group and the single drug group, which may be related to an insufficient sample size. These results are consistent with a previous study [39]. At the same time, the results of the subgroup analysis according to course of treatment showed that, regardless of how the treatment process and dosage form change, the combined drug group showed significant improvement in the ALT normalization rate, undetectable serum HBV-DNA rate, HBeAg seroconversion rate, and loss of serum HBeAg rate, compared with the rates observed in the single drug group.

However, there were still some limitations to this analysis. The thirty-two trials included had shortcomings in methodology, and their quality evaluations indicated potential bias. For example, most of the included studies did not provide detailed explanations regarding the blinding of outcome assessment, allocation concealment, blinding of participants and personnel, and other bias reports, which directly affected the strength of evidence and reduced the quality and reliability of the included literature. Subgroup analysis showed that the significant improvement of undetectable serum HBV-DNA rate in KS combined with ADV or ETV group was seen in less than one year. Potential publication bias still existed. Furthermore, most of the studies were performed in the Chinese subcontinent, and the influence of geographical and genetic factors could not be ruled out in this systematic review. Therefore, improvements in the methodological quality of clinical research on CHB are still needed in the future. We still need to strictly design more reasonable, high-quality, large-scale and multicentre RCTs to further verify the curative effect and safety of KS combined with ADV or ETV in the treatment of CHB to guide the clinical prescription of medications more reliably and accurately.

## 5. Conclusions

This systematic review and meta-analysis indicated that, on the basis of the RCTs currently available, compared with the use of a single drug, the combinations of KS with ADV or ETV not only improved the rates of ALT normalization, loss of serum HBeAg, undetectable serum HBV-DNA, HBeAg seroconversion, and loss of serum HBsAg but also decreased serum HBV-DNA levels to some extent. In addition, no serious adverse reactions occurred in any of the included studies. Furthermore, the combination drug group and single drug group did not show a significant difference in the incidence of side effects or ALT normalization. In summary, KS combined with ADV or ETV could be a safe and beneficial treatment for humans that could improve the efficacy of CHB treatment. However, we still need to carry out more high-quality, large-scale, multicentre RCTs worldwide to verify the efficacy of this treatment and guide the clinical prescription of medications more reasonably.

## Figures and Tables

**Table 1:** Characteristics of included studies on KS combined with ADV or ETV for the treatment of CHB.

Author, year	Cases C/T	Age (years) range, mean	Gender: male/female	Interventions C/T	Dosage and route of administration	Course of treatment	Adverse events	Outcome measures
Li et al., 2020	90/90	22–42, 32.3 ± 8.7	127/53	C: ETV T: ETV + KS	ETV: 0.5 mg/d, po, qd KS: 0.2 g/time, po, tid	6 months	NR	①③④

Xia et al., 2020	51/51	C: 18–63, 48 ± 9 T: 20–65, 48 ± 9	C: 29/22 T: 27/24	C: ETV T: ETV + KS	ETV: 0.5 mg/d, po, qd KS: 0.2 g/time, po, tid	1 year	√	①④⑦

Zhao and Li, 2019	62/62	C: 19–57, 32.56 ± 7.38 T: 21–58, 31.43 ± 6.79	C: 44/18 T: 45/17	C: ETV T: ETV + KS	ETV: 0.5 mg/d, po, qd KS: 0.2 g/time, po, tid	48 weeks	√	①④⑤⑥⑦

Wang et al., 2017	50/50	32.34 ± 7.44	C: 30/20 T: 32/18	C: ETV T: ETV + KS	ETV: 0.5 mg/d, po, qd KS: 0.2 g/time, po, tid	48 weeks	NR	①②⑤⑥⑦

Kang, 2016	40/40	47.5 ± 4.7	43/37	C: ETV T: ETV + KS	ETV: 0.5 mg/d, po, qd KS: 0.2 g/time, po, tid	48 weeks	0	①②③

Zhang et al., 2016	104/112	C: 21–57 T: 22–58	C: 57/47 T: 63/49	C: ETV T: ETV + KS	ETV: 0.5 mg/d, po, qd KS: 0.2 g/time, po, tid	48 weeks	NR	①②③④⑤⑦

Sun, 2016	53/53	39.5 ± 4.7	79/27	C: ETV T: ETV + KS	ETV: 0.5 mg/d, po, qd KS: 0.2 g/time, po, tid	12 months	√	①②③④⑤⑦

Fang et al., 2014	30/60	16–65	NR	C: ADV T: ADV + KS	ADV: 10 mg/d, po, qd KS: 0.2 g/time, po, tid	24 months	√	①②③

Qian and Hu, 2014	48/44	C: 35.13 ± 7.45 T: 34.27 ± 6.23	C: 41/7 T: 35/9	C: ADV T: ADV + KS	ADV: 10 mg/d, po, qd KS: 0.2 g/time, po, tid	1 year	√	①②③④⑤⑦

Ren et al., 2014	48/52	47.5 ± 4.8	53/47	C: ETV T: ETV + KS	ETV: 0.5 mg/d, po, qd KS: 0.2 g/time, po, tid	48 weeks	√	①②③

Wu, 2013	42/40	C: 36.6 ± 8.6 T: 36.9 ± 6.9	C: 24/18 T: 23/17	C: ADV T: ADV + KS	ADV: 10 mg/d, po, qd KS: 0.3 g/time, po, tid	12 months	0	①③④

Xu and Liang, 2013	40/40	16–65	NR	C: ADV T: ADV + KS	ADV: 10 mg/d, po, qd KS: 0.2 g/time, po, tid	48 weeks	√	①②③

Zhang, 2013	38/38	46.8 ± 3.9	39/37	C: ADV T: ADV + KS	ADV: 10 mg/d, po, qd KS: 0.2 g/time, po, tid	48 weeks	√	①③④

Gong and Sheng, 2013	50/52	C: 35.1 ± 4.5 T: 32.2 ± 5.6	C: 41/9 T: 42/10	C: ETV T: ETV + KS	ETV: 0.5 mg/d, po, qd KS: 0.2 g/time, po, tid	1 year	√	①②③④⑤⑦

Zhang et al., 2013	50/59	C: 31.5 (26, 37) T: 33.0 (28, 44)	C: 29/21 T: 38/21	C: ETV T: ETV + KS	ETV: 0.5 mg/d, po, qd KS: 0.4 g/time, po, tid	48 weeks	√	①②③④

Shen, 2013	36/34	16–48	42/28	C: ADV T: ADV + KS	ADV: 10 mg/d, po, qd KS: 0.2 g/time, po, tid	48 weeks	NR	①③④

Zhao, 2013	104/104	C: 32.8 ± 4.7 T: 31.3 ± 4.6	C: 68/36 T: 72/32	C: ETV T: ETV + KS	ETV: 0.5 mg/d, po, qd KS: 0.2 g/time, po, tid	1 year	NR	①②③④

Yin, 2013	50/50	C: 42.8 ± 8.1 T: 44.1 ± 9.2	C: 30/20 T: 29/21	C: ADV T: ADV + KS	ADV: 10 mg/d, po, qd KS: 0.2 g/time, po, tid	12 months	√	①②③④⑤⑦

Hu and Sun, 2012	52/54	C: 30.63 ± 10.53 T: 29.48 ± 8.27	C: 34/18 T: 38/16	C: ADV T: ADV + KS	ADV: 10 mg/d, po, qd KS: 0.2 g/time, po, tid	52 weeks	√	①②③④⑤

Lv et al., 2011	50/54	C: 33.5 T: 32.5	C: 39/11 T: 42/12	C: ADV T: ADV + KS	ADV: 10 mg/d, po, qd KS: 0.3 g/time, po, tid	9 months	0	①②③⑥

Yan et al., 2011	62/70	C: 32 ± 6 T: 32 ± 6	C: 49/13 T: 52/18	C: ADV T: ADV + KS	ADV: 10 mg/d, po, qd KS: 0.15 g/time, po, tid	52 weeks	√	①④

Yang et al., 2011	40/40	22–48 (37)	48/32	C: ADV T: ADV + KS	ADV: 10 mg/d, po, qd KS: 0.2 g/time, po, tid	12 months	√	①④⑦

Zhang, 2011	34/30	C: 35 T: 33	C: 23/11 T: 18/12	C: ADV T: ADV + KS	ADV: 10 mg/d, po, qd KS: 0.2 g/time, po, tid	3 years	√	①②③④

Cheng 2011	44/48	34 ± 5.8	63/29	C: ETV T: ETV + KS	ETV: 0.5 mg/d, po, qd KS: 0.2 g/time, po, tid	48 weeks	NR	①②③④

Yin and Ni, 2011	30/30	NR	NR	C: ETV T: ETV + KS	ETV: 0.5 mg/d, po, qd KS: 0.2 g/time, po, tid	48 weeks	NR	①②④

Shen, 2010	34/38	16–65	42/30	C: ADV T: ADV + KS	ADV: 10 mg/d, po, qd KS: 0.2 g/time, po, tid	12 months	NR	①②③④⑥

Zhang and Hu, 2010	32/46	C: 31.3 ± 7.9 T: 32.5 ± 8.3	C: 22/10 T : 32/14	C: ADV T: ADV + KS	ADV: 10 mg/d, po, qd KS: 0.2 g/time, po, tid	12 months	√	①③④

Zhou, 2010	115/115	42.5 ± 4.8	168/62	C: ADV T: ADV + KS	ADV: 10 mg/d, po, qd KS: 0.3 g/time, po, tid	48 weeks	√	①②③④⑥

Shao and Zhang, 2010	44/48	34 ± 5.8	63/29	C: ETV T: ETV + KS	ETV: 0.5 mg/d, po, qd KS: 0.2 g/time, po, tid	48 weeks	NR	①②③④

Zhang, 2009	40/40	18–55 (38)	60/20	C: ADV T: ADV + KS	ADV: 10 mg/d, po, qd KS: 0.2 g/time, po, tid	1 year	NR	①②③

Wei et al., 2008	39/33	15–65	43/29	C: ADV T: ADV + KS	ADV: 10 mg/d, po, qd KS: 0.2 g/time, po, tid	48 weeks	NR	①②③④

Liu et al., 2007	30/34	16–65	NR	C: ADV T: ADV + KS	ADV: 10 mg/d, po, qd KS: 0.2 g/time, po, tid	12 months	0	①②③④⑥

① Undetectable serum HBV-DNA rate, ② loss of serum HBeAg rate, ③ ALT normalization rate, ④ HBeAg seroconversion rate, ⑤ serum HBV-DNA level, ⑥ loss of serum HBsAg rate, and ⑦ serum ALT levels. 0, reported with no cases. Kushenin: KS; ADV: adefovir dipivoxil; ETV: entecavir; CHB: chronic hepatitis B; NR: not reported; C: control groups; T: trial groups; HBeAg: hepatitis B e antigen; HBsAg: hepatitis B surface antigen; ALT: alanine aminotransferase.

**Table 2:** Subgroup analysis on treatment period in patients with CHB treated with KS combined with ADV or ETV.

Outcomes	Trials	Participants	Treatment period	Control group	Trials group	Heterogeneity		RR (95% CI)	*P*-value
						*P*	*I* ^2^ (%)		
Undetectable serum HBV-DNA rate	16	1751	Less than one year	548/848	751/903	0.06	38	1.28 (1.21, 1.36)	<0.00001
	16	1592	One year or more	448/784	588/808	0.02	46	1.28 (1.19, 1.37)	<0.00001

Loss of serum HBeAg rate	13	1425	Less than one year	259/684	454/741	0.66	0	1.62 (1.45, 1.80)	<0.00001
	10	986	One year or more	124/487	222/499	0.98	0	1.74 (1.45, 2.08)	<0.00001

HBeAg seroconversion rate	10	1197	Less than one year	157/590	273/607	0.88	0	1.69 (1.45, 1.97)	<0.00001
	15	1504	One year or more	135/736	308/768	0.99	0	2.18 (1.83, 2.60)	<0.00001

ALT normalization rate	13	1411	Less than one year	509/678	632/733	0.51	0	1.11 (1.06, 1.17)	<0.00001
	13	1334	One year or more	475/659	574/675	0.0006	65	1.14 (1.05, 1.24)	<0.00001

KS: Kushenin; ADV: adefovir dipivoxil; ETV: entecavir; CHB: chronic hepatitis B; HBeAg: hepatitis B e antigen; HBsAg: hepatitis B surface antigen; ALT: alanine aminotransferase.

## Data Availability

The data used to support the findings of this study are available from the corresponding author upon reasonable request.

## Abbreviations

CHB: Chronic hepatitis B
TCM: Traditional Chinese medicine
KS: Kushenin
ADV: Adefovir dipivoxil
ETV: Entecavir
CNKI: China National Knowledge Infrastructure
RCTs: Randomized controlled trials
HBeAg: Hepatitis B e antigen
SMD: Standardized mean difference
HBsAg: Hepatitis B surface antigen
ALT: Alanine aminotransferase.

## Supplementary Materials

Supplementary Materials Supplementary Table 1: adverse events of each study in detail.
