# Supplementary material for: Kushenin Combined with Adefovir Dipivoxil or Entecavir for Chronic Hepatitis B: A Systematic Review and Meta-Analysis
Source: Evid Based Complement Alternat Med. 2021 Feb 25;2021:8856319. doi: 10.1155/2021/8856319 (PMC7932775; doi:10.1155/2021/8856319)
Supplement: Supplementary Materials — Supplementary Table 1: adverse events of each study in detail. [file 8856319.f1.docx]

**Supplementary Table 1. Adverse events of each study in details.**

| **Author, year** | **Adverse events** |
| --- | --- |
|  |  |
| Li et al. 2020 | NR |
| Xia et al. 2020 | C: One case was dizzy and one case was nausea and vomiting.  T: 0 |
| Zhao and Li 2019 | C: There were 2 cases of influenza like syndrome, 3 cases of digestive system reaction and 2 cases of skin reaction.  T: There were 2 cases of influenza like syndrome, 2 cases of digestive system reaction and 1 case of skin reaction. |
| Wang et al. 2017 | NR |
| Kang 2016 | 0 |
| Zhang et al. 2016 | NR |
| Sun 2016 | A few cases had nausea, poor appetite, slight stomach discomfort and dizziness. |
| Fang et al. 2014 | Some cases with rash |
| Qian and Hu 2014 | A very small number of patients had a transient slight increase in serum creatinine levels and a decrease in blood phosphorus levels at the initial stage of treatment, which had no obvious effect on the treatment. |
| Ren et al. 2014 | 2 cases in control group and 1 case in the experimental group had mild dizziness. |
| Wu 2013 | 0 |
| Xu and Liang 2013 | C: 1 case had proteinuria and 2 cases had mild diarrhoea.  T: 2 cases had upper abdominal discomfort and mild diarrhoea; 2 cases had fatigue and chest tightness. However, all these symptoms were tolerated and relieved after symptomatic treatment. |
| Zhang 2013 | C: 1 case with light diarrhoea and 1 case with proteinuria.  T: 2 cases had mild diarrhoea and abdominal discomfort, 1 case had chest tightness and weakness. Both groups of patients could tolerate their symptoms, and the group recovered without medication. |
| Gong and Sheng 2013 | 2 cases in control group and 1 case in the experimental group had mild dizziness and abdominal discomfort. |
| Zhang et al. 2013 | 16 cases in control group and 24 cases in the experimental group had mild side effects. |
| Shen 2013 | NR |
| Zhao 2013 | NR |
| Yin 2013 | C: 2 cases had slight fatigue and diarrhoea, and 1 case had slight creatinine elevation.  T: One patient had dizziness and skin tumour itching, and one patient had abdominal discomfort. |
| Hu and Sun 2012 | C: 2 cases had nausea, abdominal distension and discomfort in the liver area.  T: 4 cases had diarrhoea, headache, loss of appetite and fatigue. However, all these symptoms were tolerated and relieved after symptomatic treatment. |
| Lv et al. 2011 | 0 |
| Yan et al. 2011 | C: 1 case had slight epigastric discomfort.  T: 2 cases had slight epigastric discomfort after taking the medicine, and 1 case had slight nausea. |
| Yang et al. 2011 | There were several cases of nausea and abdominal distension, which all improved after symptomatic treatment. |
| Zhang 2011 | 2 cases in control group and 3 cases in the experimental group had mild nausea, anorexia, and upper abdominal discomfort. |
| Cheng 2011 | NR |
| Yin and Ni 2011 | NR |
| Shen 2010 | NR |
| Zhang and Hu 2010 | 2 cases in control group and 3 cases in the experimental group had mild nausea, anorexia, and upper abdominal discomfort. |
| Zhou 2010 | These two groups mainly had nausea, anorexia, acid regurgitation and other upper gastrointestinal symptoms. 26 cases in the experimental group had a transient reduction in cholinesterase. |
| Shao and Zhang 2010 | NR |
| Zhang 2009 | NR |
| Wei et al. 2008 | NR |
| Liu et al. 2007 | 0 |

Notes: C = control group, T = treatment group, CHB = chronic hepatitis B, NR = not report.
